# The Rise of Tunnel Endoscopic Surgery: A Case Report and Literature Review

**DOI:** 10.1155/2012/847640

**Published:** 2012-09-04

**Authors:** Mingyan Cai, Jinzhong Chen, Pinghong Zhou, Liqing Yao

**Affiliations:** ^1^Endoscopy Center and Endoscopy Research Institute, Zhongshan Hospital, Fudan University, 180 Fenglin Road, Shanghai 200032, China; ^2^Department of Gastrointestinal Endoscopy, No.1 Hospital Affiliated to Xiamen University, Fujian, Xiamen 361003, China

## Abstract

There has been booming interest in natural orifice transluminal surgery since it was first described. Several techniques first developed for the safe transluminal access now derive into independent endoscopic surgical procedures. In this paper, we describe a case treated by a novel procedure by submucosal tunnelling technique and provide a literature review of the rise of tunnel endoscopic surgery.

## 1. Introduction

 Tunnel endoscopy is a novel approach which was initially developed for the purpose of establishing an access for natural orifice transluminal endoscopic surgery (NOTES) [1–3]. Meanwhile, it brought a great idea for endoscopic procedures by using the submucosal tunnel as an operating space. The emerge of peroral endoscopic myotomy (POEM) for treating esophageal achalasia marked the rising of a new branch of therapeutic endoscopy [4]. Our group defines it as tunnel endoscopic surgery which includes several novel procedures utilizing a submucosal tunnel as an operating space. We innovate a new method by tunnelling technique for treating submucosal tumors originating from muscularis propria [5]. Herein we report a case operated by this novel method: submucosal tunnelling endoscopic resection (STER). 

## 2. Case Report 

A 55-year-old male patient presented with a chief complaint of discomfort behind his sternum when swallowing. A submucosal tumor about 1.5 cm × 2.0 cm was found at middle esophagus (26 cm from the incisors) by gastroscopy. The endoscopic ultrasound revealed the tumor originated in the muscularis propria. After obtaining the institutional review board approval and informed consent, STER was arranged for this patient and was performed by an experienced endoscopist (PHZ).

**Figure 1:**
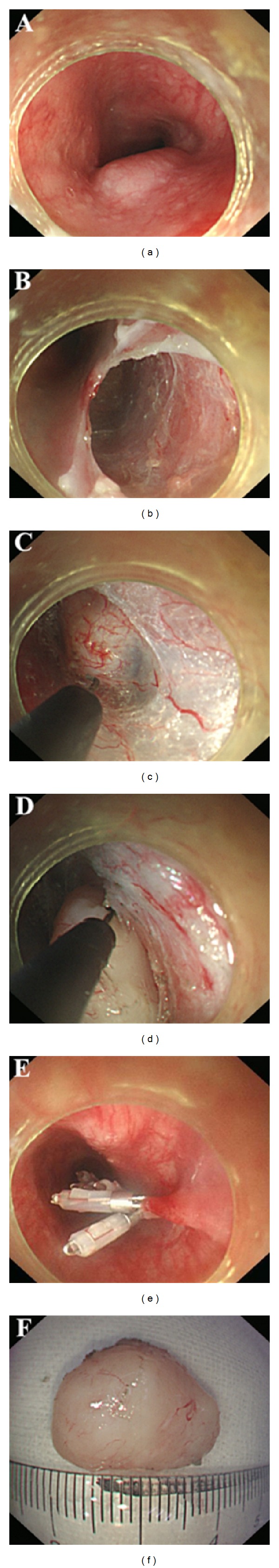
The endoscopic tunnelling submucosal resection procedure. (a) A submucosal tumor was found in esophagus (26 cm from incisors). (b) After mucosal injection, a submucosal tunnel (about 5 cm long) was created by ESD technique. (c) The tumor was successfully exposed in the tunnel. (d) Tumor resection was being carried out in the tunnel. (e) The mucosal incision site was sealed by hemostatic clips. (f) The resected specimen was 1.5 cm × 2.0 cm.

The procedure was carried out under general anaesthesia with trachea intubation. It was consisted of the following steps: mucosal injection and incision, submucosal tunnelling, tumor resection, and mucosal closure (Figure 1). The equipment needed was the same as that of standard endoscopic submucosal resection (ESD) [6–8]. The mucosal injection and incision point were chosen at approximately 5 cm above the lesion site. After mucosal injection, a 2 cm mucosal incision was made by a hook knife. Then, a submucosal tunnel was created from the incision site to 1-2 cm below the lesion as long as it was fully exposed by ESD technique. The tumor was completely dissected from the muscularis layer while the adventitia being intact. After the specimen retrieval and the hemostasis in the submucosal tunnel, the mucosal incision site was close by metallic clips. The whole procedure took 30 minutes. 

**Figure 2:**
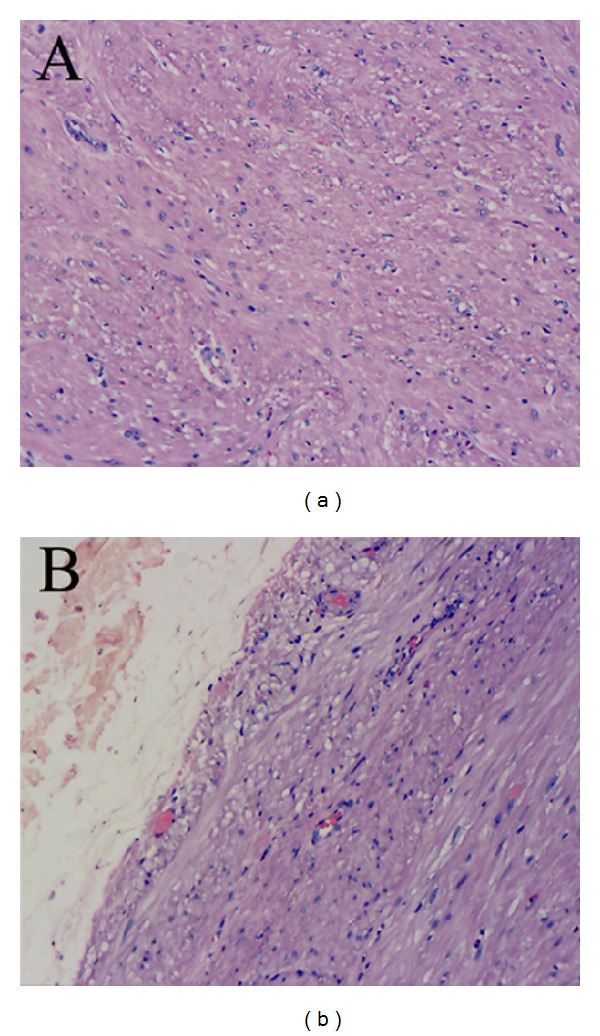
Pathology of the resected specimen. The pathology showed a leiomyoma (a) and clear margins (b) (magnifying ×200).

**Figure 3:**
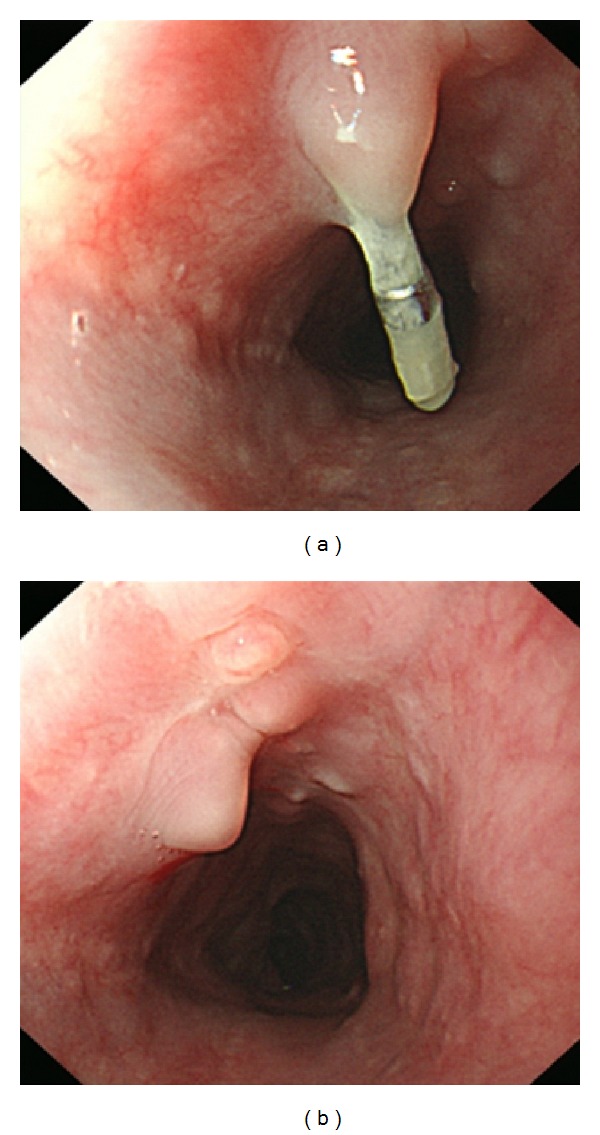
Follow-up esophagoscopy. On postoperative month 2, the follow-up esophagoscopy demonstrated a well-healed mucosa.

The patient felt no pain after procedure though a subcutaneous emphysema and a minor left pneumothorax were observed on CT scan on postoperative day 1. No further intervention was necessary. The patient was given full liquid diet on postoperative day 2. The symptom of dysphagia relieved immediately. He was discharged uneventfully. The pathology result showed an esophageal leiomyoma with clear margins (Figure 2). The follow-up esophagoscopy revealed a well-healed mucosa (Figure 3).

## 3. Discussion and Literature Review

This case demonstrates that the application of tunnelling technique is feasible for the en bloc resection of upper gastrointestinal submucosal tumors from deep layer. Our preliminary data of large scales is in publication [5]. Herein we review the development and the new emerging procedures of tunnel endoscopic surgery.

## 4. Tunnel Endoscopic Surgery in Animal Model 

A blossomed interest has been aroused in NOTES since it was first reported in 2004 [9]. Tunnel endoscopy, also referred as “submucosal endoscopy with mucosal flap safety valve” (SEMF) technique [2, 10–12] or “self-approximating transluminal access technique” (STAT) [1, 13], was developed for the safe access of NOTES in porcine model in 2007. The two independent groups shared the similar thoughts by creating an ST from the wall of natural orifice; however, the methodology of tunnelling technique they used varied a little from each other. In SEMF technique, the authors created submucosal tunnel by high-pressure carbon dioxide (CO_2_) injection and balloon dissection [2, 3]. The shortcoming of this method is that high-pressure CO_2_ injection and balloon dissection may create an excessive large submucosal tunnel that partial necrosis of the overlying free mucosa occurred in three of four pigs [3]. Besides, a fluid injection was still needed for the prevention of gas escape. While in STAT, a fluid cushion of normal saline was made, and submucosal tunnel was created by the sharp and blunt dissection of a grasping forcep and the leading edge of the endoscopy itself [1]. The evidence to date suggests that STAT allows safe and reliable translumenal access and the removal of small-to-moderate-sized (<8 × 3 cm) specimens during NOTES  procedures in animal models [14].  A comparative study of four different transgastric access showed that an extended submucosal tunnel yielded the best leak resistance, which is superior to standard transgastric access methods and rival handsewn interrupted stitches [15]. However, neither balloon dissection nor forcep dissection could avoid the risk of bleeding when tunnelling. The application of endoscopic submucosal dissection (ESD) technique in tunnel endoscopy optimized this transluminal access [16].

Actually, the attempt of tunnel endoscopic surgery also dated back to 2007. That means tunnel endoscopy was not only developed as an access for NOTES but also considered as an intervention treatment. Submucosal endoscopic esophageal myotomy was depicted as a novel experimental approach for the treatment of achalasia. A submucosal saline lift was created approximately 5 cm above the lower esophageal sphincter (LES), and a small nick was made in the mucosa in order to facilitate the introduction of a dilating balloon. After dilation, the scope was introduced over the balloon into the submucosal space and advanced toward the now visible fibers of the LES. The circular layer of muscle was then cleanly incised using an electrocautery knife in a distal-to-proximal fashion, without complications [17].

## 5. Tunnel Endoscopic Surgery in Human 

The milestone of tunnel endoscopic surgery in clinical application is POEM for treating esophageal achalasia. After submucosal injection, the endoscopy enters the submucosal space and begins submucosal tunnelling. A long submucosal tunnel is created to 3 cm distal to the gastroesophageal junction (GEJ). Then, endoscopic myotomy is carried out from 3 cm below the mucosal incision point in a proximal to distal manner to a total length of 10 cm. Finally the mucosal incision site is closed by hemostatic clips [4]. In all 17 cases including 5 difficult ones (sigmoid type), the patients had a great improvement in clinical manifestations and manometric findings. No procedure related serious complication encountered. The success of the procedure is the very first attempt for applying tunnel endoscopic surgery for treating human diseases. Moreover, utilizing the submucosal space not only as a transluminal access but also as an independent surgical method marks the beginning of tunnel endoscopic surgery as a new branch of interventional endoscopy. 

Inspired by the success in POEM and other tunnel endoscopic applications in NOTES, we innovated the novel procedure based on submucosal tunnelling technique. In the reported case, STER was carried out by creating a submucosal tunnel from 5 cm distal to 1-2 cm proximal to the submucosal tumor by a hook knife to provide enough operation space for the next step: endoscopic dissection. The tumor was dissected carefully by an insulated-tipped knife from the muscularis propria layer, keeping the adventitia intact as possible. If a perforation occurs inevitably, the overlying mucosa can provide a seal for the wall defect. Finally, the mucosal entry point was closed with hemostatic clips. It is a promising procedure for several reasons. (a) For submucosal tumors originating from deeper layers of the gut wall, perforation is no longer a fearful issue during the procedure. Perforation is only a procedure-related event and can be successfully managed after tumor resection. After the withdrawal of endoscopy from the submucosal tunnel, the overlying free mucosa serves as a sealant flap over the myomectomy site and immediately holds the air escaping into the mediastinum or abdomen even in an unclipped ex vivo model [1]. Thus, it provides abundant time for mucosa closure. (b) An extended submucosal tunnel of 5 cm showed the best leak resistance compared with standard transgastric access methods such as linear incision and balloon dilation [15]. (c) The equipment used in STER is the same as that of ESD, while ESD is an established method for treating early mucosal carcinoma or submucosal tumors. 

## 6. Conclusion

The development of tunnel endoscopy and its variation of surgical procedures are inspiring for both gastroenterologists and surgeons. We believe that the future of flexible endoscopy is not only operating in the natural orifice but also within and outside the gastrointestinal wall. The latter needs more innovation and courage for the endoscopists.
